# Transhepatectomy combined with arterial chemoembolization and transcatheter arterial chemoembolization in the treatment of hepatocellular carcinoma: a clinical prognostic analysis

**DOI:** 10.1186/s12876-023-02886-1

**Published:** 2023-09-05

**Authors:** Xin Liu, Haodong Li, Fei Wang, Ke Su, Bingsheng He, Jie He, Jiaqi Zhong, Yunwei Han, Zhenjiang Li

**Affiliations:** 1 Department of Oncology, Affiliated Hospital of Southwest Medical University, Luzhou, China; 2 Department of Radiophysics and Technology, Shandong First Medical University (Shandong Academy of Medical Sciences), Shandong Institute of Cancer Prevention and Treatment (Shandong Cancer Hospital), Jinan, China; 3 Graduate Department of Shandong First Medical University (Shandong Academy of Medical Sciences), Jinan, China; 4 Department of General Surgery, Luxian People’s Hospital, Luzhou, China; 5 Shandong Institute of Cancer Prevention and Treatment (Shandong Cancer Hospital), Department of Radiotherapy, Shandong First Medical University (Shandong Academy of Medical Sciences), Jinan, China

**Keywords:** Hepatocellular carcinoma, Transarterial chemoembolization, Clinical efficacy, Hepatectomy, Interventional radiology

## Abstract

**Background:**

The prognosis of patients undergoing hepatectomy combined with transarterial chemoembolization (TACE) and TACE alone was examined in order to better understand the role of hepatectomy in the treatment of hepatocellular carcinoma (HCC). In this work, we also created a model and investigated the variables influencing overall survival (OS) in HCC patients.

**Methods:**

Retrospective analysis of 1083 patients who received TACE alone as the control group and 188 patients who received TACE after surgery in a total of 1271 HCC patients treated with LR + TACE or TACE at three third-class hospitals in China. It was done using the Propensity Score Matching (PSM) technique. The differences in OS between the two groups were compared, and OS-influencing factors were looked at. The main endpoint is overall survival. In this study, the COX regression model was used to establish the nomogram.

**Results:**

The median OS of the LR + TACE group was not attained after PSM. The median OS for the TACE group was 28.8 months (95% CI: 18.9–38.7). The median OS of the LR + TACE group was higher than that of the TACE group alone, indicating a significant difference between the two groups (χ^2^ = 16.75, *P* < 0.001). While it was not achieved in the LR + TACE group, the median OS for patients with lymph node metastases in the TACE group alone was 18.8 months. The two groups differed significantly from one another (χ^2^ = 4.105, *P* = 0.043). In patients with distant metastases, the median OS of the LR + TACE treatment group was not achieved, and the median OS of the TACE group alone was 12.0 months. The difference between the two groups was sizable (χ^2^ = 5.266, *P* = 0.022). The median OS for patients with PVTT following PSM was 30.1 months in the LR + TACE treatment group and 18.7 months in the TACE alone group, respectively. The two groups differed significantly from one another (χ^2^ = 5.178, *P* = 0.023); There was no discernible difference between the two groups in terms of median overall survival (OS), which was 30.1 months for patients with lymph node metastasis and 19.2 months for those without (*P* > 0.05); Regarding the median OS for patients with distant metastases, which was not achieved and 8.5 months, respectively, there was a significant difference between the two groups (χ^2^ = 5.759, *P* = 0.016). We created a new nomogram to predict 1-, 2-, and 3-year survival rates based on multiple independent predictors in COX multivariate analysis. The cohort's C-index is 0.705. The area under the curve (AUC value) for predicting 1-, 2-, and 3-year survival rates were shown by the subject operating characteristic (ROC) curve linked to the nomogram to be 0.730, 0.728, and 0.691, respectively.

**Conclusions:**

LR + TACE can increase OS, delay tumor recurrence, and improve prognosis in HCC patients when compared to TACE alone. Additionally, the nomogram we created does a good job of forecasting the 1-year survival rate of hepatocellular carcinoma.

## Background

Liver cancer is one of the most typical malignant tumors [1]. Hepatocellular carcinoma (HCC), the sixth most prevalent cancer in the world, is responsible for 85%-90% of primary liver cancer [2]. Hepatic artery chemoembolization (TACE), radiotherapy, immunity, and targeted therapy are examples of palliative care for HCC, whereas tumor ablation, hepatectomy, and liver transplantation are examples of radical therapy for the disease [3, 4]. Early stage HCC is defined as HCC with a physical strength score (PS) of 0 and a Child–Pugh score of A or B, as well as a maximum diameter of a single cancer node or the maximum diameter of two cancer nodules combined that is less than 3 cm. Early stage HCC is usually treated with surgical resection as the first option [5]. Recent research indicates that patients with HCC are more likely to survive after hepatectomy, but because the disease frequently returns and patient long-term survival rates are suboptimal, postoperative adjuvant therapy is crucial for reducing postoperative HCC recurrence [6]. Drug-eluting beads transarterial chemoembolization (DEB-TACE) and traditional transarterial chemoembolization (cTACE) are two types of transarterial chemoembolization (TACE) [7]. The liver is supplied by two blood vessels, the hepatic artery and the portal vein, and the hepatic malignant tumor is primarily supplied by the hepatic artery, so TACE can deliver chemotherapy drugs directly to the tumor feeding artery while protecting the healthy liver tissue supplied by the portal vein [8]. When used in TACE, embolic agents can stop blood flow, allow the infusion of cytotoxic drugs to kill tumor cells, and significantly slow blood vessel growth and invasion [9]. TACE has a high safety factor because it is a minimally invasive procedure. A study found that patients with advanced HCC who received TACE had a median survival time of 19.9 months [10]. Adjuvant TACE following radical resection of liver cancer has been shown to improve both the recurrence-free survival time (RFS) and overall survival (OS) after hepatectomy in numerous studies [11]. Few studies have been reported, despite the fact that TACE has been used in adjuvant therapy. We conducted a retrospective study of patients treated with radical hepatectomy combined with TACE and TACE alone in order to better understand the prognostic variations and influencing factors of these two surgical procedures. In order to clarify TACE's role in the treatment of HCC, the main objective of this study is to compare the prognosis of patients who undergo HCC surgery in combination with TACE and TACE alone. Additionally, we developed a model and looked into the influencing factors of OS in patients with hepatocellular carcinoma in this study.

## Methods

In this study, patients with hepatocellular carcinoma who underwent hepatectomy plus TACE or TACE alone were retrospectively analyzed across multiple centers. A total of 1271 HCC patients from three tertiary hospitals in China who had surgery or TACE treatment between June 2016 and July 2021 were gathered to participate in the review. The retrospective study was carried out with the patients' informed consent and was approved by the institutional review boards of Shandong Cancer Hospital (SDTHEC2022012021), Luxian People's Hospital (2,022,032), and the affiliated hospital of Southwest Medical University (KY2020254). The investigation has been registered with the China Clinical Trials Registry under the registration number ChiCTR2100051057. Each patient who is eligible for this study complies with the following criteria:

Inclusion criteria:

- HCC diagnosis by cytology or histology;
- Patients aged 18 and above;
- Child–Pugh level A or B;
- Eastern United States Cancer Cooperation (Eastern Cooperative Oncology Group, ECOG) score: 0–1;
- No severe major organ dysfunction;
- Technically consistent with intra-arterial treatment, no extensive arteriovenous shunt.
- After hepatectomy, only TACE adjuvant therapy is used.

Exclusion criteria:

- History of iodine allergy and/or sensitivity to contrast agents;
- History of other malignant tumors;
- There is any serious physical and/or mental disorder;
- Insufficient available data provided;
- Irreversible renal function impairment, defined as serum creatinine level ≥ 200 mol/L;
- Have serious complications, such as severe cardiac or renal insufficiency or blood coagulation dysfunction.

### Surgical treatment

The mode of operation is traditional laparotomy. The surgical resection was performed under a general anesthetic. For anatomical hepatectomy, the resection margin was at least 1 cm. The location of the tumor and any relevant liver conditions also affect the particulars of the hepatectomy.

### TACE treatment

In this study, 188 patients received postoperative auxiliary TACE, while 1083 people received TACE alone. In the post-PSM, 225 patients were in the TACE alone group and 133 patients were in the postoperative auxiliary TACE group. Postoperative combination TACE therapy was administered without contraindications one month after hepatectomy. TACE alone is used to treat patients who either won't undergo surgery or have medical reasons not to. Lipiodol, gelatin sponge particles, and anti-cancer drugs are slowly injected while being monitored by X-rays. The dosage of antineoplastic drugs, lipiodol, and embolic material tablets were chosen for TACE treatment based on the size and level of tumor invasion. If the patient's condition deteriorates or new lesions appear, TACE treatment can be repeated. Before drafting the treatment plan, the patient is assessed by the hospital's HCC expert group.

### Evaluation and follow-up

One month after treatment, the expert team evaluated the immediate effects of surgical resection or TACE using imaging analysis. For the past two years, patients have undergone routine clinical reexaminations. After that, they have undergone liver function tests, AFP tests, thoracic and abdominal dynamic enhanced CT scans, and finally reexaminations every three to six months until tumor recurrence. The objective of the study is to analyze the patient's OS.

### Statistical analysis

The IBM SPSS (Version 25.0) statistical program (IBM Corporation, Armonk, NY, USA) was used to conduct the statistical analysis. The counting data is expressed using frequency. The Propensity Score Matching (PSM) method was used to reduce the selection deviation and confounding effects caused by the dissimilar covariable distribution between the LR + TACE group and the TACE group. PSM detection factors included sex, age, Child–Pugh grade, number and size of tumors, alpha-fetoprotein, AFP, TACE times, BCLC stage, hepatitis type, drinking history, other tissue invasion, and previous treatment. The Kaplan–Meier logarithmic rank test (log-rank test) was used to compare OS between groups. The Cox proportional hazard regression model was used to examine the potential OS influencing factors. Based on independent predictors and prognostic factors, the "RMS" package in R software generates a nomogram of prediction and prognosis, respectively. P 0.05 is the cutoff for statistical significance.

## Results

(Table 1) provides summary statistics and descriptive data broken down by therapy. Between June 2016 and July 2021, 1271 people who met the requirements underwent neoadjuvant therapy. The propensity score was calculated using a multivariate logistic regression model, and the closest neighbor matching method was used for 1:2 matching. 358 patients (133 treated with LR + TACE and 225 with TACE alone) had comparable tumor characteristics.

**Table 1:** Baseline characteristics of the patients before and after PSM

	Before PSM			After PSM		
Variable	LR plus TACE	TACE	*P*	LR plus TACE	TACE	*P*
patients	188	1083		133	225	
Male sex	157 (83.5)	922 (85.1)	0.566	116 (87.2)	193 (85.88)	0.702
Age ≥ 60 years	68 (36.2)	451 (41.6)	0.159	51 (38.3)	86 (38.2)	0.981
Child–Pugh B	17 (9.0)	277 (25.6)	< 0.001	15 (11.3)	37 (16.4)	0.18
Tumor number	1.75 ± 0.90	1.18 ± 0.39	< 0.001	1.88 ± 0.86	1.63 ± 0.70	0.789
Tumor size, cm	5.8 (3.2–9.3)	6.5 (3.8–9.9)	< 0.001	6.6 (4.1–10.0)	6.5 (4.0–9.7)	0.34
Serum AFP ≥ 400 ng/ml	61 (32.4)	524 (48.4)	< 0.001	52 (39.1)	88 (39.1)	0.998
ALP level ≥ 125 U/L	48 (25.5)	668 (61.7)	< 0.001	46 (34.6)	98 (43.6)	0.094
Platelet count ≥ 100 × 109/L	142 (75.5)	765 (70.6)	0.171	93 (69.9)	166 (73.8)	0.431
ALT level ≥ 40 U/L	84 (44.7)	599 (55.3)	0.007	59 (44.4)	113 (50.2)	0.283
leukocyte ≥ 4 × 10^9^/L	165 (87.8)	869 (80.2)	0.014	112 (84.2)	197 (87.6)	0.374
Number of TACE	1 (1–8)	1 (1–8)		1 (1–7)	1 (1–7)	
BCLC						0.574
A	65 (34.6)	123 (11.4)		34 (25.6)	54 (24.0)	
B	63 (33.5)	162 (15.0)		42 (31.6)	62 (27.6)	
C	60 (31.9)	798 (73.7)		57 (42.9)	109 (48.4)	
Etiology						
HBV	115 (61.2)	624 (57.6)	0.362	78 (58.6)	138 (61.3)	0.616
HCV	4 (2.1)	28 (2.6)	0.712	4 (3.0)	6 (2.7)	0.850
Alcohol	71 (37.8)	445 (41.1)	0.392	49 (36.8)	83 (36.9)	0.993
Portal vein invasion	33 (17.6)	416 (38.4)	< 0.001	30 (22.6)	55 (24.4)	0.685
Lymph node metastasis	29 (15.4)	585 (54)	< 0.001	29 (21.8)	58 (25.8)	0.397
Extrahepatic metastases	10 (5.3)	248 (22.9)	< 0.001	10 (7.5)	19 (8.4)	0.756
Lung	4 (2.1)	154 (14.2)		4 (3.0)	8 (3.6)	
Bone	2 (1.1)	55 (5.1)		2 (1.5)	6 (2.7)	
other	4 (2.1)	70 (6.5)		4 (3.0)	7 (3.1)	
Previous therapy	28 (14.9)	282 (26.0)	0.001	28 (21.1)	47 (20.9)	0.972
Systemic therapy	3 (1.6)	35 (3.2)		3 (2.3)	4 (1.8)	
Liver resection	17 (9.0)	122 (11.3)		17 (12.8)	28 (12.4)	
Radiotherapy						
TACE	14 (7.4)	178 (16.4)		14 (10.5)	29 (12.9)	
RFA	6 (3.2)	26 (2.4)		6 (4.5)	7 (3.1)	

*Abbreviations*: *PSM* Propensity score matching, *AFP* Alpha fetoprotein, *ALP* Alkaline phosphatase, *ALT* Alanine aminotransferase, *PVTT* Portal vein tumor thrombus, *HBV* Hepatitis B virus, *HCV* Hepatitis C virus, *GKR* Gamma knife radiosurgery, *TACE* Transcatheter arterial chemoembolization, *RFA* Radiofrequency ablation

**Fig. 1:**
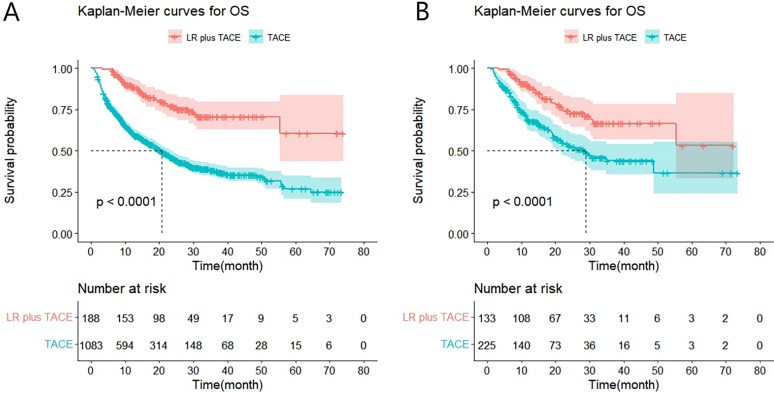
Kaplan–Meier curves for OS in patients with advanced HCC treated with LR + TACE or TACE before (**A**) and after (**B**) PSM

The median OS of the LR + TACE group had not been reached before PSM. The overall median OS was 24.4 months (95% CI: 21.2–27.6), compared to 20.6 months (95% CI: 18.0–23.2) for the TACE alone group. Between the two groups, there was a significant difference (χ^2^ = 58.14, *P* < 0.001). The LR + TACE group's median OS was not attained after PSM. The overall median OS was 48.9 months (95% CI: 29.1–68.6), compared to a median OS of 28.8 months (95% CI: 18.9–38.7) for the TACE group alone. The LR + TACE group's median OS was higher than that of the TACE group alone, indicating a significant difference between the two groups (χ^2^ = 16.75, *P* < 0.001) (Fig. 1A, B).

**Fig. 2:**
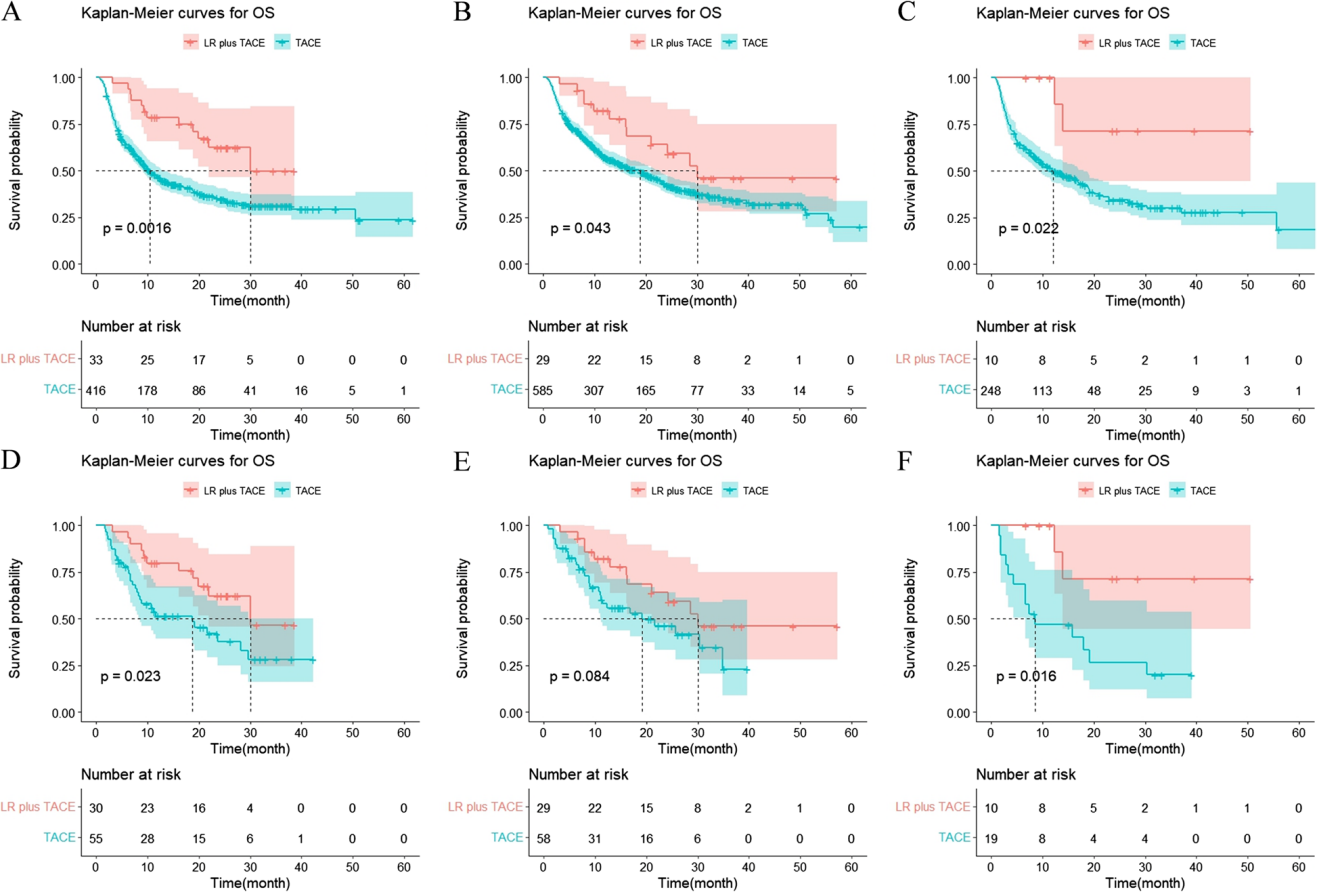
Overall survival curves for the patients with portal vein tumor thrombus PVTT (**A**), lymph node metastasis (**B**), and extrahepatic metastases (**C**) who received LR + TACE and TACE are shown before PSM. Overall survival curves for the patients with PVTT (**D**), lymph node metastasis (**E**), and extrahepatic metastases (**F**) who received LR + TACE and TACE are shown after PSM

Subgroup analysis was performed on patients who had lymph node metastases, extrahepatic distant metastases, and portal vein tumor thrombus (PVTT). In the unmatched cohort, the median OS for PVTT patients treated with LR + TACE was 30.1 months, compared to 10.5 months (95% CI: 8.68–12.33) for patients treated with TACE alone. Between the two groups, there was a significant difference (χ^2^ = 9.987, *P* = 0.002). Patients with lymph node metastasis treated with LR + TACE did not reach the median OS compared to those treated with TACE alone, and those treated with TACE alone had a median OS of 18.8 months [95% CI: 14.95–22.65], which was statistically different from the other group (χ^2^ = 4.105, *P* = 0.043). There was a significant difference between the two groups, with the median OS of patients with distant metastases receiving LR + TACE treatment not being achieved and the median OS of patients receiving TACE alone being 12.0 months [95%CI: 7.60–16.4] (χ^2^ = 5.266, *P* = 0.022). In the matched cohort, the median OS for PVTT patients treated with LR + TACE was 30.1 months, compared to 18.7 months (95% CI: 7.6–29.8) for patients treated with TACE alone. The difference between the two groups was sizable (χ^2^ = 5.178, *P* = 0.023). Patients with lymph node metastases who received LR + TACE had a median OS of 30.1 months, compared to 19.2 months for those who received TACE alone [95%CI: 5.5–32.9]. Between the two groups, there was no discernible change (*P* > 0.05). In contrast to patients treated with TACE alone, patients treated with LR + TACE did not achieve the median OS of patients with distant metastases, whereas the median OS for patients treated with TACE alone was 8.5 months [95%CI: 0.0–20.1]. Significant differences existed between the two groups (χ^2^ = 5.759, *P* = 0.016) (Fig. 2).

Before PSM, the 1-year, 2-year, and 4-year LR + TACE groups had OS rates of 89.0%, 76.4%, and 63.7%, compared to 61.1%, 45.4%, and 34.5% for the TACE group alone (*P* < 0.001). Following PSM, the 1-, 2-, and 4-year LR + TACE group's OS rates were 89.2%, 73.0%, and 57.4%, compared to 68.1%, 52.7%, and 37.1% for the TACE group (*P* < 0.001). The 5-year OS rates between the two groups before and after PSM were significantly different (*P* < 0.001) from one another.

A multivariate study of prognostic variables revealed a substantial correlation between overall survival and AFP ≥ 400 ng/ml and ALP ≥ 125U/L in both univariate and multivariate Cox regression analysis after PSM. The AFP content (*P* = 0.013), ALP content (*P* = 0.003), and treatment method (*P* = 0.001) were three independent variables that had an impact on HCC. There are two separate risk variables for HCC: AFP content [HR = 1.604(95% CI: 1.107–2.325)] and ALP content [HR = 1.782 (95% CI: 1.217–2.607)]. The risk of death increases with the concentration of AFP and ALP. HCC patients with LR and TACE therapy have a better prognosis than those with TACE alone [HR = 0.416 (95% CI: 0.275–0.628)] (Table 2).

**Table 2:** Univariate and multivariate Cox regression analysis of overall survival after PSM

	Univariable Cox regression			Multivariable Cox regression		
Variable	HR	95%CI	*P*	HR	95%CI	*P*
Sex (male/female)	0.868	0.520–1.449	0.588			
Age (≥ 60/ < 60 years)	0.725	0.499–1.054	0.092			
Child–Pugh class (B/A)	1.757	1.122–2.750	0.014*	1.454	0.905–2.336	0.122
Number of tumor (≥ 2/ < 2)	1.522	1.009–2.295	0.045*	1.510	0.936–2.435	0.091
Tumor diameter (≥ 5/ < 5 cm)	1.585	1.075–2.335	0.020*	0.829	0.528–1.303	0.417
AFP (≥ 400/ < 400 ng/ml)	1.795	1.262–2.553	0.001*	1.604	1.107–2.325	0.013*
ALP (≥ 125/ < 125 U/L)	1.937	1.363–2.752	< 0.001*	1.782	1.217–2.607	0.003*
Platelet (< 100,000/ ≥ 100,000/μL)	0.899	0.614–1.317	0.586			
ALT (≥ 40/ < 40U/L)	1.597	1.118–2.283	0.010*	1.383	0.952–2.009	0.089
leukocyte (< 4000/ ≥ 4000/μL)	1.311	0.764–2.249	0.326			
BCLC			< 0.001*			0.321
A	1.000			1.000		
B	1.276	0.706–2.308	0.420	0.577	0.234–1.422	0.232
C	2.826	1.691–4.721	< 0.001*	0.549	0.248–1.213	0.138
HBV (positive/negative)	0.897	0.627–1.284	0.554			
HCV (positive/negative)	0.486	0.120–1.966	0.312			
Alcoholism (positive/negative)	0.998	0.695–1.433	0.990			
Portal vein invasion (yes/no)	1.952	1.344–2.836	< 0.001*	1.173	0.661–2.081	0.586
Lymph node metastasis (yes/no)	1.756	1.208–2.552	0.003*	1.126	0.600–2.115	0.712
Extrahepatic metastases (yes/no)	1.942	1.149–3.284	0.013*	1.484	0.721–3.057	0.284
Previous therapy (yes/no)	0.435	0.256–0.738	0.002*	0.625	0.354–1.106	0.106
Treatment (LR plus TACE/TACE)	0.442	0.295–0.661	< 0.001*	0.416	0.275–0.628	< 0.001*

*Abbreviations*: *HR* Hazard ratio, *BCLC* Barcelona Clinic Liver Cancer, *AFP* Alpha-fetoprotein, *ALP* Alkaline phosphatase, *ALT* Alanine transaminase, *HBV* Hepatitis B virus, *HCV* Hepatitis C virus, *LR* Liver resection, *TACE* Transcatheter arterial chemoembolization

*P < 0.05

**Fig. 3:**
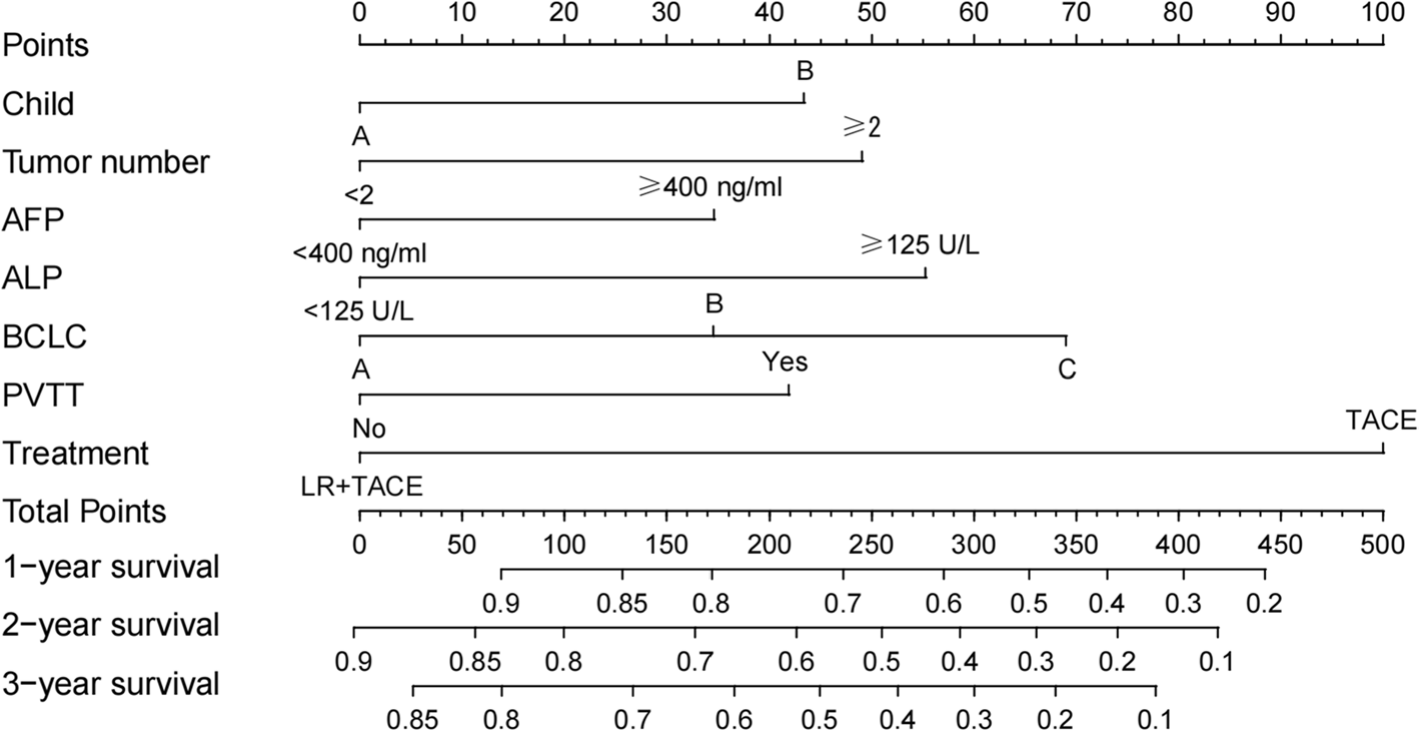
Nomogram for predicting OS from HCC patients

**Fig. 4:**
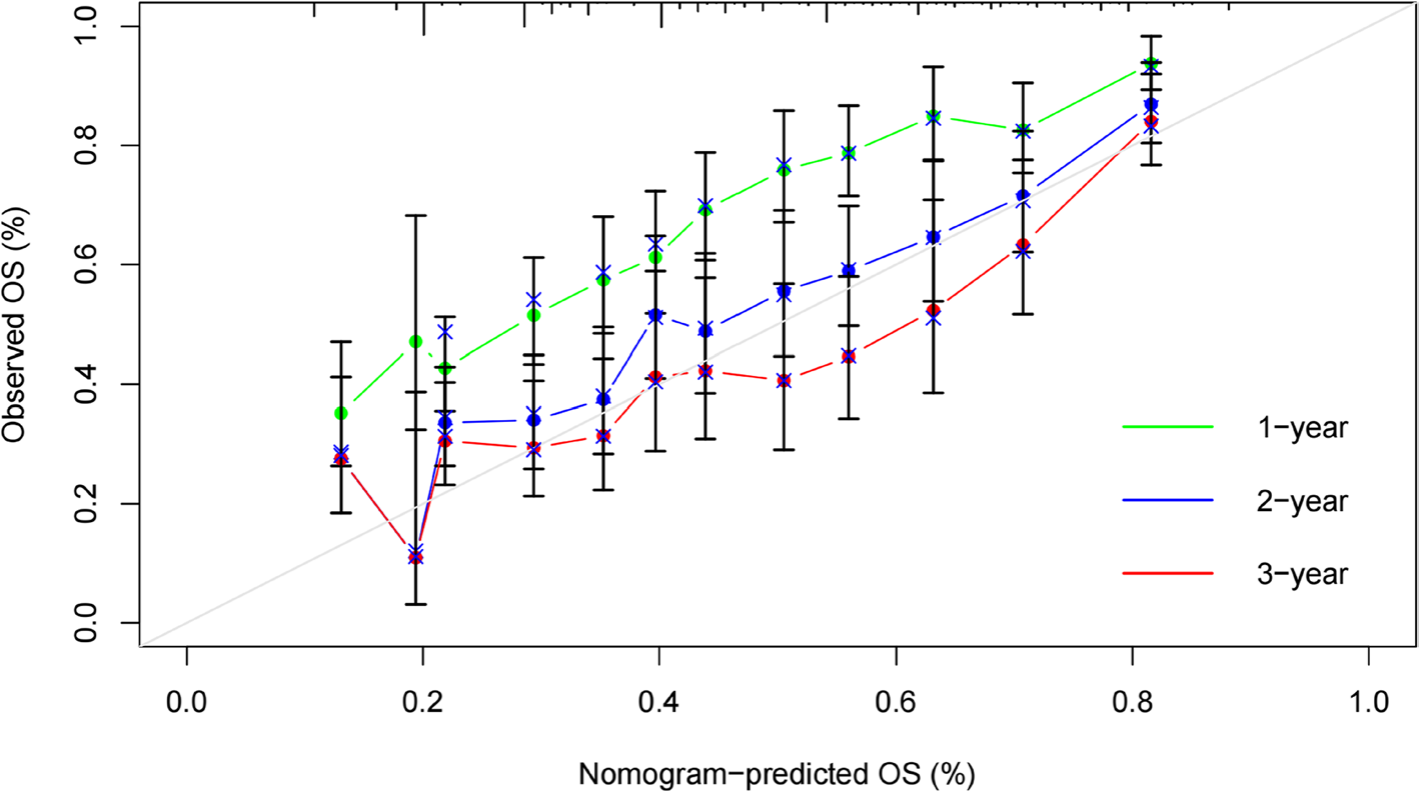
Calibration curves for predicting 1-, 2-, and 3-year survival rates

**Fig. 5:**
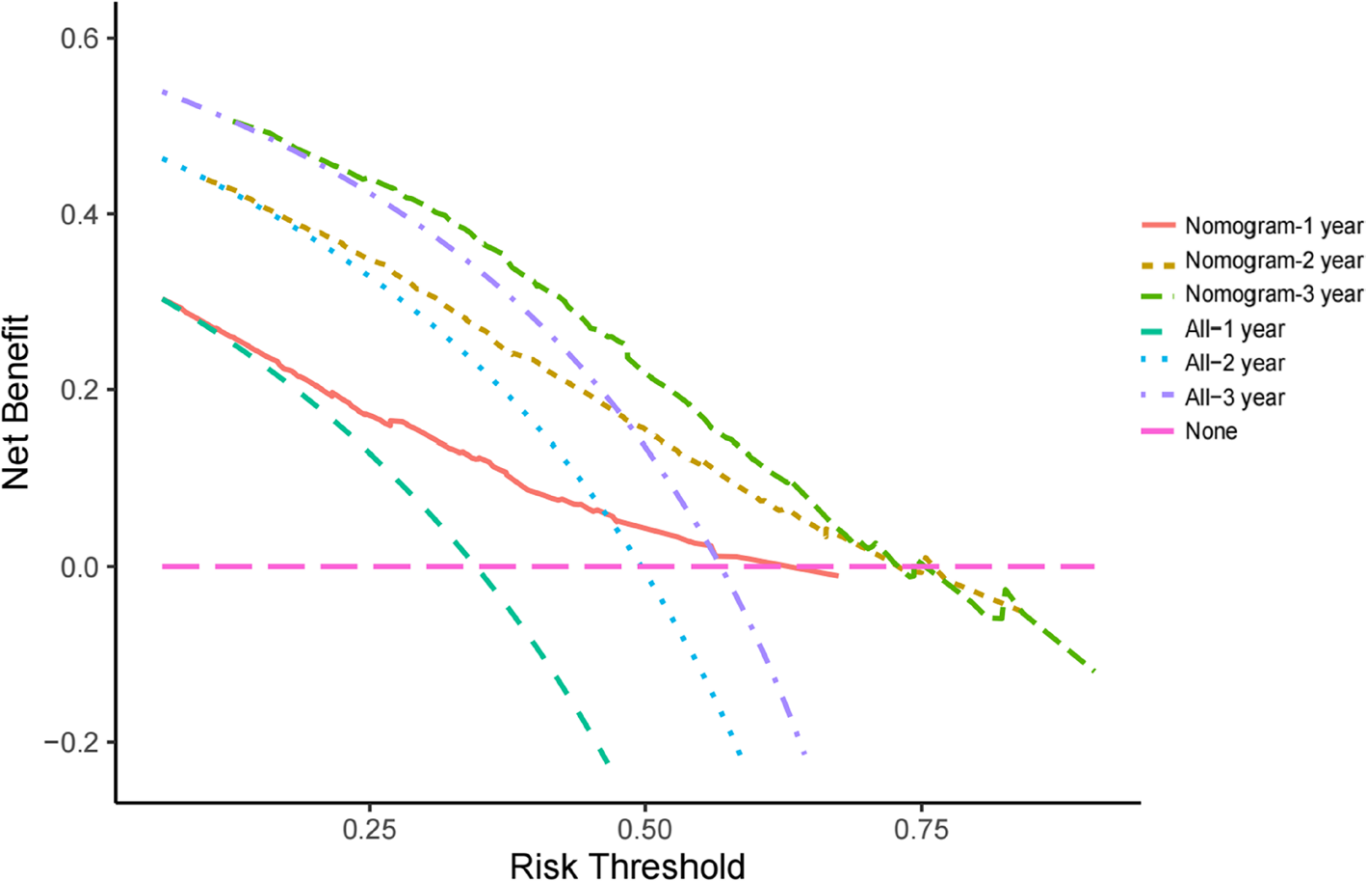
Decision curve analyses (DCA) curves of 1-, 2-, and 3-year survival rates

**Fig. 6:**
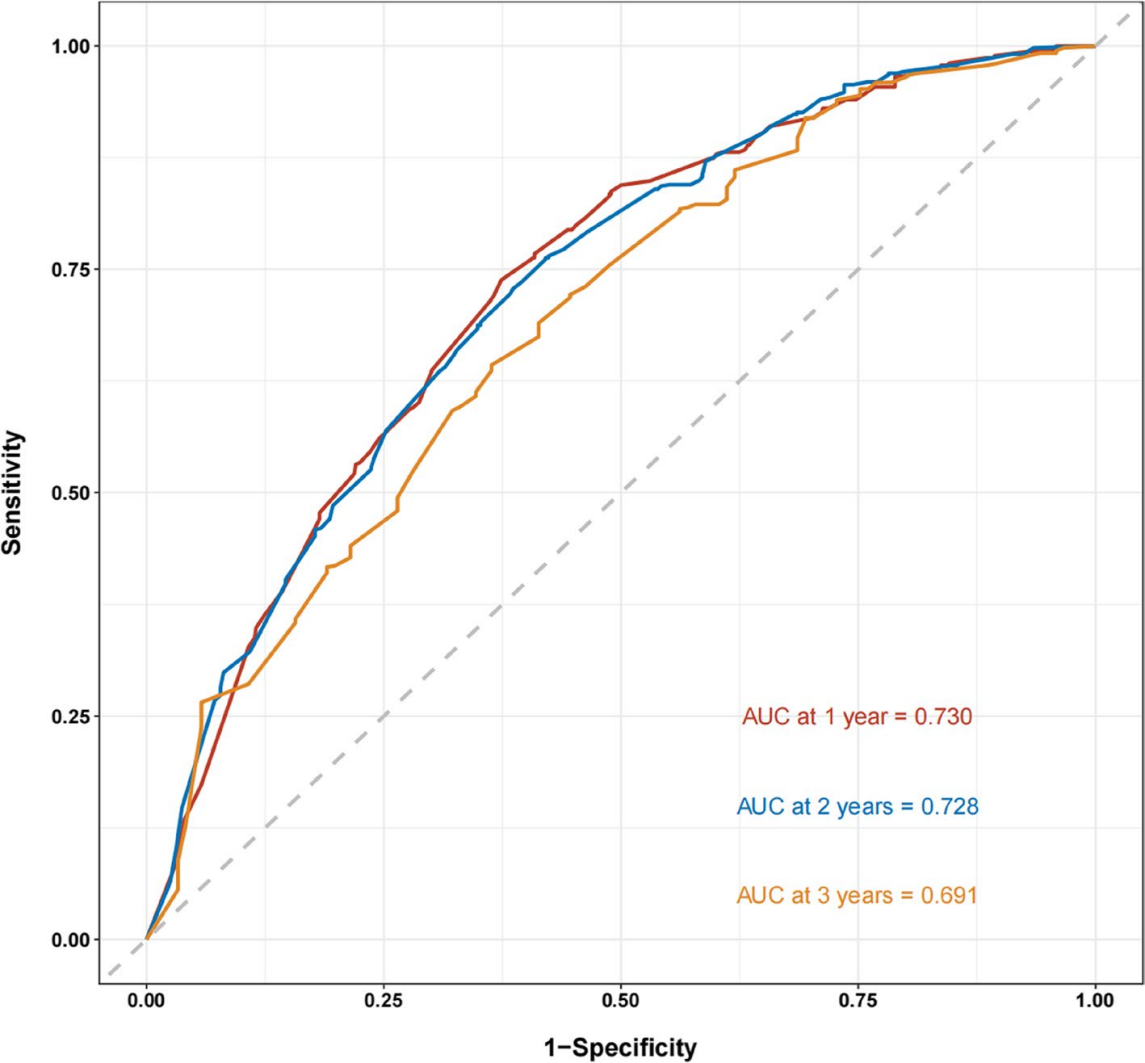
Nomogram-related operating characteristics curve (ROC) curves for survival

Nineteen predictive factors were examined using univariate analysis prior to PSM. According to the findings, 12 predictors were linked to OS in hepatocellular carcinoma patients. AFP, ALP, BCLC stage, portal vein invasion, and treatment mode were independent predictors of OS in patients with HCC, according to multivariate COX regression analysis (Table 3). A nomogram was developed for OS risk assessment in patients with hepatocellular carcinoma based on several independent OS-related variables (Fig. 3). The cohort's C-index is 0.705. The calibration curve demonstrates that there is good agreement between the observed survival rates and those predicted by the nomogram for the first, second, and third years (Fig. 4). In terms of clinical application, the DCA curves of the 1-, 2-, and 3-year survival rates exhibit good promise (Fig. 5). As DCA [12] shows, "all" and "none" mean to assume that all patients are alive and dead, respectively. Use Time ROC package of R software to draw ROC curve and calculate AUC value [13]. According to the nomogram-related ROC curve (Fig. 6), the AUC values for predicting the 1-, 2-, and 3-year survival rates were 0.730, 0.728, and 0.691, respectively.

**Table 3:** Univariate and multivariate Cox regression analysis of overall survival before PSM

	Univariable Cox regression			Multivariable Cox regression		
Variable	HR	95%CI	*P*	HR	95%CI	*P*
Sex (male/female)	1.008	0.803–1.266	0.944			
Age (≥ 60/ < 60 years)	0.775	0.655–0.917	0.003			
Child–Pugh class (B/A)	1.808	1.509–2.166	< 0.001*	1.351	1.116–1.634	0.002*
Number of tumor (≥ 2/ < 2)	1.945	1.557–2.430	< 0.001*	1.360	1.061–1.743	0.015*
Tumor diameter (≥ 5/ < 5 cm)	1.829	1.504–2.225	< 0.001*	1.082	0.871–1.344	0.478
AFP (≥ 400/ < 400 ng/ml)	1.629	1.384–1.917	0.001*	1.236	1.041–1.466	0.015*
ALP (≥ 125/ < 125 U/L)	2.108	1.775–2.505	< 0.001*	1.426	1.176–1.729	< 0.001*
Platelet (< 100,000/ ≥ 100,000/μL)	1.095	0.914–1.311	0.326			
ALT (≥ 40/ < 40U/L)	1.393	1.181–1.643	< 0.001*	1.078	0.906–1.284	0.396
leukocyte (< 4000/ ≥ 4000/μL)	1.193	0.961–1.481	0.109			
BCLC			< 0.001*			0.037*
A	1.000			1.000		
B	2.241	1.526–3.291	< 0.001*	1.571	1.022–2.413	0.039*
C	3.797	2.715–5.311	< 0.001*	1.689	1.087–2.626	0.020*
HBV (positive/negative)	1.028	0.872–1.211	0.746			
HCV (positive/negative)	0.757	0.417–1.374	0.360			
Alcoholism (positive/negative)	1.106	0.938–1.303	0.232			
Portal vein invasion (yes/no)	1.997	1.695–2.353	< 0.001*	1.379	1.129–1.684	0.002*
Lymph node metastasis (yes/no)	1.570	1.334–1.848	< 0.001*	0.978	0.788–1.215	0.843
Extrahepatic metastases (yes/no)	1.731	1.437–2.085	< 0.001*	1.188	0.971–1.452	0.094
Previous therapy (yes/no)	0.789	0.649–0.958	0.017*	0.860	0.704–1.052	0.143
Treatment (LR plus TACE/TACE)	0.311	0.227–0.428	< 0.001*	0.465	0.333–0.649	< 0.001*

*Abbreviations*: *HR* Hazard ratio, *BCLC* Barcelona Clinic Liver Cancer, *AFP* Alpha-fetoprotein, *ALP* Alkaline phosphatase, *ALT* Alanine transaminase, *HBV* Hepatitis B virus, *HCV* Hepatitis C virus, *LR* Liver resection, *TACE* Transcatheter arterial chemoembolization

*P < 0.05

## Discussion

According to studies, neoadjuvant TACE regimens for hepatocellular carcinoma can be used as a palliative treatment for unresectable recurrent hepatocellular carcinoma or as a way to shrink tumors and improve resectability in cases where they are currently untreatable [14, 15]. TACE also appears to be a successful treatment for patients who have recurrence following radical hepatectomy [16]. TACE is frequently administered to patients who have a rich compensatory collateral circulation, incomplete occlusion of the main portal vein, or recanalization of a portal vein stent. Following local injection of cytotoxic agents, tumor vessel embolization with lipophilic or particulate embolic agents can result in ischemic necrosis of the tumor, postpone the swift elution of chemotherapy, increase local concentrations of anticancer agents in the target area, and maximize anticancer drug efficacy. Reduce systemic toxicity as well [17–19]. However, following TACE therapy, the tumor is exposed to hypoxia, which increases the risk of HCC recurrence and metastasis by upregulating the expression of vascular endothelial growth factor, tumor revascularization, and local recurrence [20]. As hepatectomy safety has increased, the OS of patients with early HCC has significantly increased. Systematic treatment can reduce the clinical stage or the tumor target area in patients with middle and late stages of HCC, allowing for drastic resection [21]. There is a significant chance of intrahepatic recurrence (up to 80%) even if the surgical resection is successful [22]. LR combined with TACE therapy may therefore be a useful strategy for patients to increase the survival rate of HCC patients.

The results of Feng et al. showed that the 3-year RFS rates for patients who received 2 or 1 adjuvant TACE treatment after HCC were 73.0% and 55.0%, respectively, and were only 29.3% in the group who underwent surgery alone. Patients who underwent an operation alone had significantly worse 3-year RFS than those who underwent one TACE adjuvant therapy after surgery (*P* = 0.024). The prognosis was better for patients who received two adjuvant TACE treatments as opposed to just one (*P* = 0.033) [23]. Because the frequency of postoperative TACE treatments varies, it is impossible to determine how frequently TACE treatments affect survival or recurrence. This is one of the study's limitations. Unresectable HCC patients who received TACE alone had 1-year and 2-year Overall Survival rates of 82.7% and 64.6%, respectively, according to Kudo et al. [24]. Qi et al. discovered that the operation group's overall survival time was significantly longer than the TACE group's (HR = 0.60, 95%CI = [0.55–0.66]), as well as the operation group's 1-, 3-, and 5-year survival rates (OR = 1.82, 95%CI = [1.56–2.14], OR = 3.09, 95%CI = [2.60–3.67], and OR = 3.48, 95%CI = [2.83–4.27]) [25]. Although neither surgery nor TACE by themselves can result in a higher survival rate, postoperative TACE can clearly significantly increase the survival of patients with HCC. In order to reduce the deviation caused by the two groups' baseline features being confused, PSM was used in this investigation. The rate of OS varied significantly between the LR + TACE group and the TACE group. After a significant hepatectomy, patients may benefit from a postoperative TACE combination to delay tumor recurrence and improve their chances of living a disease-free life.

TACE may be beneficial for patients with advanced liver cancer because it effectively treats both local and distant liver cancer metastases without causing any obvious side effects [26]. Surgery combined with TACE may be beneficial for patients with early and medium stage HCC. In comparison to patients without PVTT, those who received TACE + LR showed a higher survival percentage [27]. The results of this study showed that the median OS of the LR + TACE group was higher than that of the TACE group in patients with PVTT, lymph node metastases, and distant metastases. Multivariate analysis revealed that ALP, AFP, and therapy were independent predictors of OS. The concentration of AFP and ALP raises the risk of death. HCC patients who received LR along with TACE had a better prognosis than those who only received TACE. According to Huang et al., patients with extensive hepatectomy and high ALP (> 81U/dL) showed significant vascular invasion and recurrence [28]. AI was used by Chicco et al. to determine the clinical factors affecting OS and predict the survival rate of HCC. The analysis identified hemoglobin, AFP, and ALP blood levels as independent prognostic factors. The prognosis gets worse as ALP and AFP concentrations rise. In adults, AFP can be elevated in about 80% of liver cancer patients; the level of serum AFP is positively correlated with the size of the liver cancer, which is a sign of high tumor invasiveness. Primary liver cancer, metastatic liver cancer, or liver abscess may cause a significant increase in ALP [29]. The findings of Liu et al. showed that among the 246 patients who had no recurrence within one month of the operation, the OS and RFS of the postoperative adjuvant TACE (LR + TACE) group were significantly better than those of the non-LR-TACE group. With LR-TACE therapy, the survival rate of HCC patients with PVTT after hepatectomy was improved [30]. It is essential to combine LR and TACE because patients who receive LR + TACE have a better prognosis than those who receive TACE alone, which is in line with the findings of this study. In this study, the tumor stage, number, size, PVTT, and extrahepatic metastasis were significant predictors of OS; however, after adjustment in the multivariate analysis, these factors had no statistically significant impact on OS. The study's small sample size and brief follow-up period may be to blame for this.

In our research, we developed a prognostic nomogram for HCC patients. By collecting data from a number of readily accessible variables on the nomogram of each liver cancer patient, the total score can be calculated. The model we developed estimates the survival rate of each patient rather than just categorizing patients into different risk categories, better mitigating the effects of heterogeneity than early prognostic indicators like BCLC, ALBI, and Child–Pugh grades. The AUC values for predicting the 1-, 3-, and 5-year survival rates in Zhang's nomogram of liver cancer were 0.645, 0.671, and 0.635, respectively [31]. The model we created for our study had a wider range of treatment options and more data, which decreased sampling deviations. The nomogram can then be used to quickly identify the risk of HCC, which can offer direction for additional clinical treatment. This nomogram performs well in predicting the survival of patients with HCC, increasing the accuracy of individualized clinical decision-making and monitoring.

## Conclusions

In conclusion, LR in conjunction with TACE is an effective treatment to improve the results of hepatectomy in patients with HCC, which may be more beneficial to patients with PVTT, to delay tumor recurrence and enhance survival rate. Additionally, the nomogram we created does a good job of forecasting the HCC 1-year survival rate.

## Data Availability

The datasets analyzed during the current study are not publicly available due to the data confidentiality requirements of the affiliated Hospital of Southwest Medical University but are available from the corresponding author upon reasonable request.
