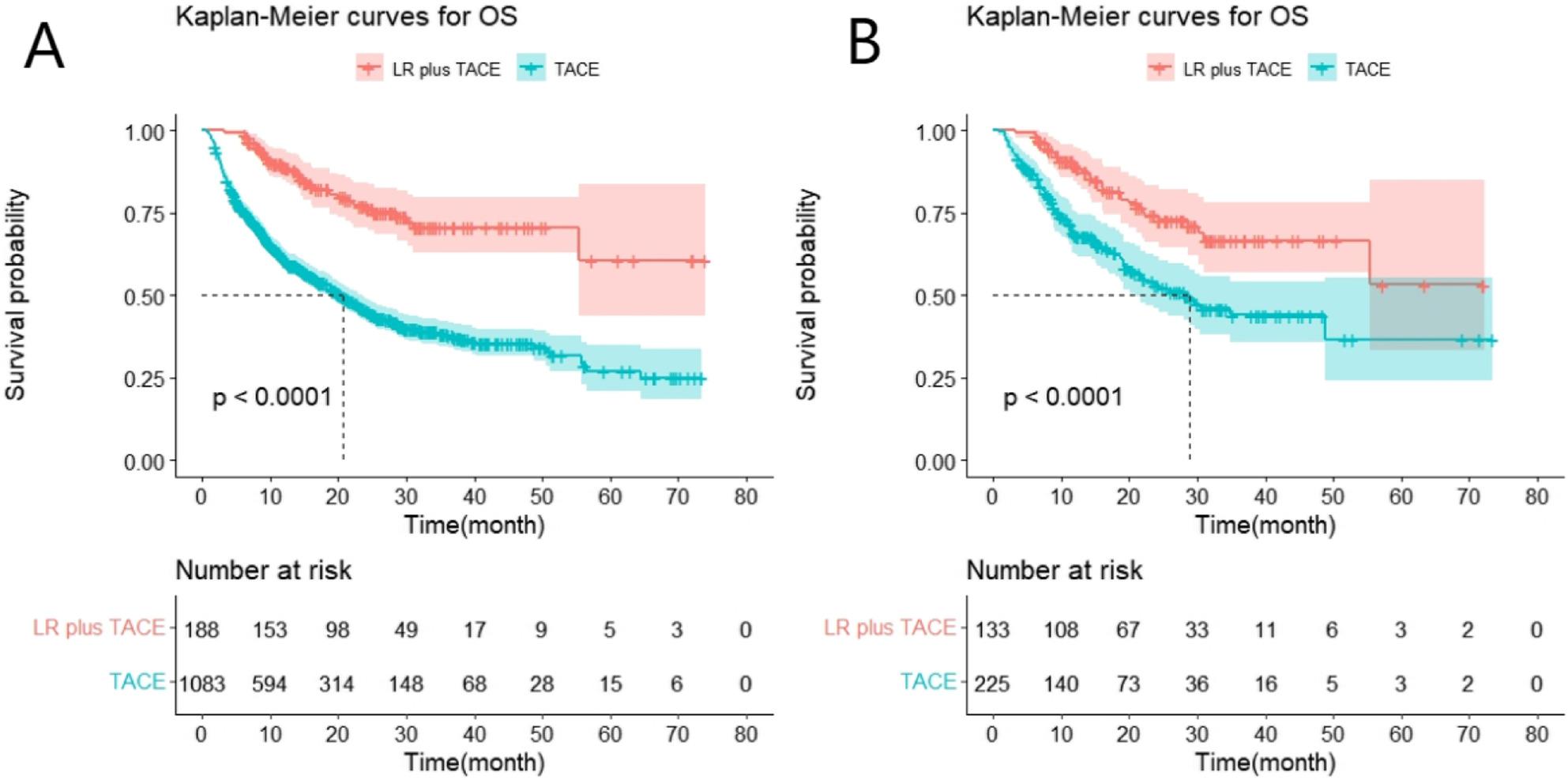# Correction: Transhepatectomy combined with arterial chemoembolization and transcatheter arterial chemoembolization in the treatment of hepatocellular carcinoma: a clinical prognostic analysis

**DOI:** 10.1186/s12876-026-04731-7

**Published:** 2026-04-09

**Authors:** Xin Liu, Haodong Li, Fei Wang, Ke Su, Bingsheng He, Jie He, Jiaqi Zhong, Yunwei Han, Zhenjiang Li

**Affiliations:** 1 Department of Oncology, Affiliated Hospital of Southwest Medical University, Luzhou, China; 2 Department of Radiophysics and Technology, Shandong First Medical University, Shandong Academy of Medical Sciences, Shandong Institute of Cancer Prevention and Treatment (Shandong Cancer Hospital), Jinan, China; 3 Graduate Department of Shandong, First Medical University (Shandong Academy of Medical Sciences), Jinan, China; 4 Department of General Surgery, Luxian People’s Hospital, Luzhou, China; 5 Department of Radiotherapy, Shandong Institute of Cancer Prevention and Treatment (Shandong Cancer Hospital, Shandong First Medical University, Shandong Academy of Medical Sciences), Jinan, China

**Correction: BMC Gastroenterol 23, 299 (2023)**

**https://doi.org/10.1186/s12876-023-02886-1**

Following publication of the original article it was reported that there was an error in Fig. 1. Panel 1B was a duplicate of Fig. 1A. The incorrect and correct Fig. 1 are given in this Correction, and the original article has been updated.

**Original Fig. 1**.


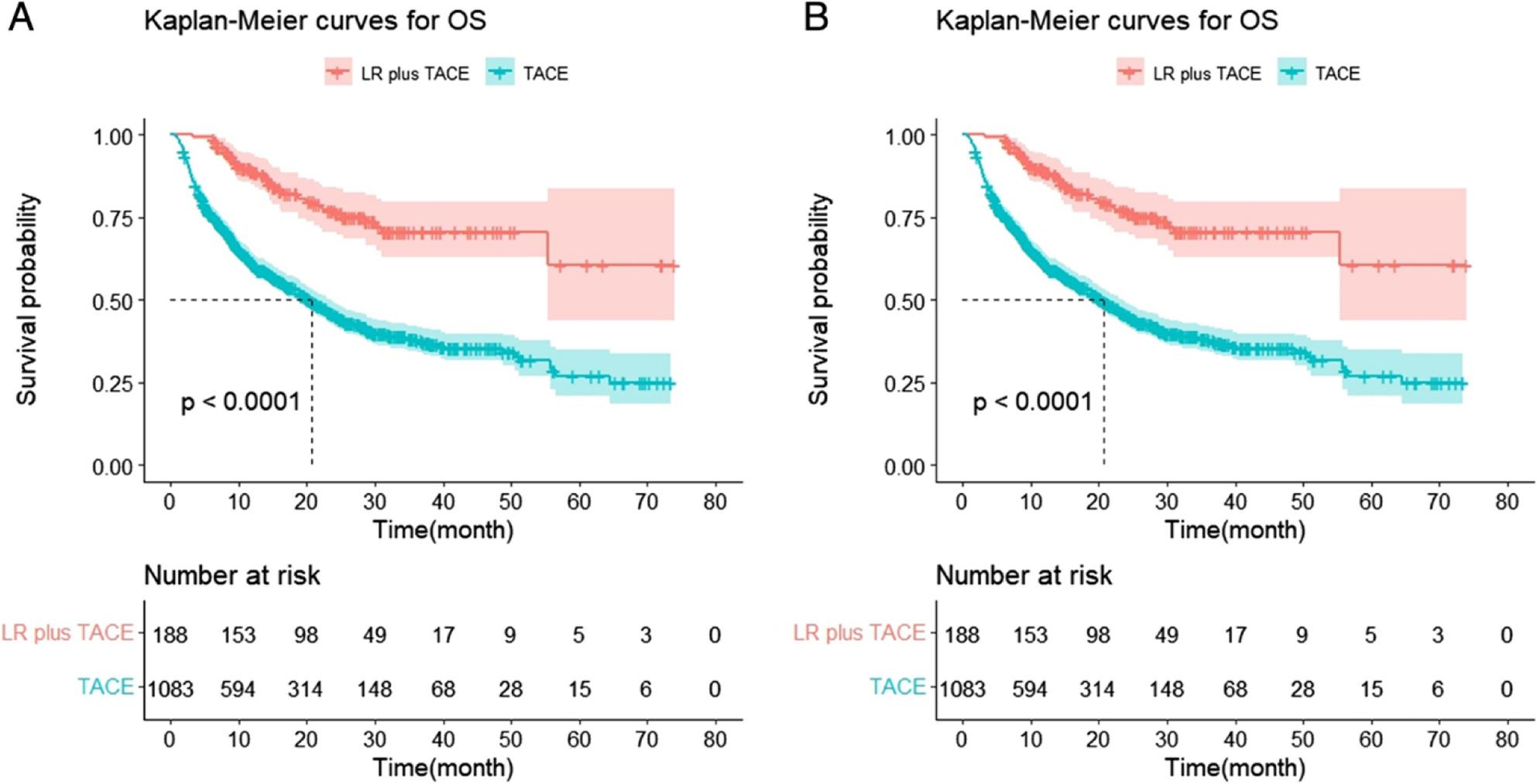


**Corrected Fig. 1**.